# Enhanced Neutralizing Antibody Titers and Th1 Polarization from a Novel *Escherichia coli* Derived Pandemic Influenza Vaccine

**DOI:** 10.1371/journal.pone.0076571

**Published:** 2013-10-18

**Authors:** David A. G. Skibinski, Brendon J. Hanson, Yufang Lin, Veronika von Messling, Andrea Jegerlehner, Jason Boon Sern Tee, De Hoe Chye, Steven K. K. Wong, Amanda A. P. Ng, Hui Yin Lee, Bijin Au, Bernett T. K. Lee, Lucia Santoso, Michael Poidinger, Anna-Marie Fairhurst, Alex Matter, Martin F. Bachmann, Philippe Saudan, John E. Connolly

**Affiliations:** 1 A*STAR Program in Translational Research on Infectious Disease, Agency for Science, Technology and Research, Singapore; 2 Singapore Immunology Network (SIgN), Agency for Science, Technology and Research, Singapore; 3 Defence Science Organisation (DSO) National Laboratories, Singapore; 4 Institut National de la Recherche Scientifique (INRS)- Institut Armand-Frappier, University of Quebec, Quebec, Canada; 5 Cytos Biotechnology AG, Zurich, Switzerland; 6 Experimental Therapeutics Centre (ETC), Agency for Science, Technology and Research, Singapore; University of Georgia, United States of America

## Abstract

Influenza pandemics can spread quickly and cost millions of lives; the 2009 H1N1 pandemic highlighted the shortfall in the current vaccine strategy and the need for an improved global response in terms of shortening the time required to manufacture the vaccine and increasing production capacity. Here we describe the pre-clinical assessment of a novel 2009 H1N1 pandemic influenza vaccine based on the *E. coli*-produced HA globular head domain covalently linked to virus-like particles derived from the bacteriophage Qβ. When formulated with alum adjuvant and used to immunize mice, dose finding studies found that a 10 µg dose of this vaccine (3.7 µg globular HA content) induced antibody titers comparable to a 1.5 µg dose (0.7 µg globular HA content) of the licensed 2009 H1N1 pandemic vaccine Panvax, and significantly reduced viral titers in the lung following challenge with 2009 H1N1 pandemic influenza A/California/07/2009 virus. While Panvax failed to induce marked T cell responses, the novel vaccine stimulated substantial antigen-specific interferon-γ production in splenocytes from immunized mice, alongside enhanced IgG2a antibody production. In ferrets the vaccine elicited neutralizing antibodies, and following challenge with influenza A/California/07/2009 virus reduced morbidity and lowered viral titers in nasal lavages.

## Introduction

Pandemics of influenza virus infection have plagued the human race for centuries and cost countless lives; the 1918 pandemic alone is estimated to have caused as many as 100 million deaths [1]. Today, we rely on vaccines to protect individuals and prevent spread of both seasonal and pandemic influenza, but the current vaccine formulations have a number of shortfalls. During the recent 2009 H1N1 pandemic a functional vaccine was produced by traditional methods but took 5 months to become available, was unaffordable for many countries, and had a maximum output of ∼900 million doses, well below the seven billion doses that would be needed to vaccinate the global population [2]. Fortunately this strain of influenza had a low mortality rate, but it served to highlight the fact that in the face of a more severe pandemic we would need a vaccine with high efficacy that was able to be produced more rapidly, in enormous quantities, and at a cost that enabled developing nations to afford the doses needed to protect their populations.

The current manufacturing process for influenza vaccines uses 6-decade-old egg-based technology which is slow and expensive [3]. A shift to mammalian cell culture as the substrate for vaccine production removes the dependence on eggs and would be more scalable, but substantial limitations remain [3]. Recombinant vaccine technologies could overcome many of these limitations and generate a higher yield of vaccine at lower cost and in a shorter time frame. As the majority of the antibodies that protect against both disease and infection are specific for the influenza virus envelope glycoprotein hemagglutinin (HA), recombinant vaccine approaches heavily focus on this protein [4]. Prominent approaches that have been applied to 2009 H1N1 pandemic vaccines include the baculovirus-based expression of HA in insect cell culture [5], the production of virus-like particles (VLPs) consisting of HA, neuraminidase (NA) and matrix M1 proteins in insect cell culture [6], *Escherichia coli* (*E. coli*) expressed flagellin-HA globular head fusion proteins [7], the viral vector based expression of HA in plants [8] and nucleic acid based vaccines [9], [10]. What is required to move forwards is the design of a vaccine that exploits the advantages of recombinant technology for its production, whilst inducing effective and robust immune responses.

Our understanding of immunity to influenza is incomplete, but it seems clear that an effective vaccine strategy must aim to induce both appropriate antibody and T cell responses. Traditionally, correlates of protection for influenza are based on antibody levels, specifically antibodies against the hemagglutinin (HA) glycoprotein which is exposed on the viral envelope [4]. However clinical studies have now demonstrated the previously underestimated role played by T cells in driving protection. A recent study demonstrates that CD4+ T cell responses correlate with less severe illness in humans [11], and in the elderly T cell responses have been found to be closer correlates of vaccine protection [12]. As result it has been proposed that influenza vaccines need to be better at inducing type-1 cytokines such as interferon-γ [12], [13]. Improved induction of such T helper type 1-associated cytokines would have a second advantage, as they encourage production of the IgG2a antibody subclass, which contributes to protection against influenza infection in mice [14]–[16].

Here we describe the testing of a novel 2009 H1N1 vaccine produced entirely in *E. coli*. The 2009 pandemic strain HA globular head domain (gH1), which contains many of the known influenza-neutralizing epitopes, is covalently linked to VLPs derived from the bacteriophage Qβ. As a result of directional chemical cross linking gH1 is displayed in a highly ordered and repetitive fashion on the surface of the VLP, which has been shown to induce a rapid and robust immune responses to the antigen [17]–[21]. In addition, *E.coli*-derived single stranded RNA (ssRNA) contained within the VLP is a ligand for toll-like receptor 7 (TLR7), and acts as a built-in adjuvant, designed to further enhance the immune response to the antigen and to promote MHC class II presentation of viral epitopes [22], [23]. When delivered together with aluminum hydroxide adjuvant, our novel Qβ-based vaccine performed as well in mice as the licensed egg-produced 2009 H1N1 pandemic vaccine (Panvax). In addition, the Qβ-based vaccine induced a potent T helper cell type 1 (Th1) response, represented by high IgG2a titers and the induction of Th1 cytokines including interferon-γ (IFNγ). Using EPIMAX, we identified the regions within both gH1 and Qβ containing the CD4^+^ T cell epitopes, and showed that the Qβ-based vaccine increases the breadth of the T cell response to HA. Furthermore we have defined the contribution of each component of the vaccine, including the adjuvant, in generating the immune response. Lastly we modeled human influenza infection using ferrets, and showed that the novel vaccine induced the same level of neutralizing antibody as the conventional vaccine, reduced morbidity and substantially decreased viral titer in nasal lavage.

## Materials and Methods

### Production of gH1-Qβ

The gH1-Qβ vaccine specific for the influenza A/California/07/2009 strain was produced as described in the accompanying paper (A. Jegerlehner, F. Zabel, A. Langer, K. Dietmeier, G. T. Jennings, P. Saudan and M. F. Bachmann, submitted for publication).

### Production of gH1-Qβ(pGlu)

To prepare gH1-Qβ VLPs devoid of packaged ssRNA, VLPs were disassembled in PBS containing 2 mM DTT, 2 mg/ml RNaseA, 1 M NaCl and purified by ion exchange and size-exclusion chromatography. The gH1-Qβ(pGlu) VLPs were reassembled in the presence of the polyanionic molecule polyglutamic acid.

### Mouse Immunizations

Female 6–8 week old BALB/c mice were immunized subcutaneously at days 0 and 28 with the indicated doses of the unadjuvanted licensed egg-produced, split virion, inactivated, A/California/07/2009 vaccine Panvax (CSL Limited, Australia), gH1, gH1-Qβ or gH1-Qβ(pGlu), formulated either with or without alum-adjuvant (Alhydrogel aluminum hydroxide adjuvant from Brenntag). For Panvax the indicated dose refers to the amount of hemagglutinin protein content of the vaccine formulation, and for gH1, gH1-Qβ or gH1-Qβ(pGlu) the indicated dose refers to the total protein content of the vaccine formulation. It should be noted that a 10 µg dose of gH1-Qβ contains 3.7 µg of gH1. This was determined by measuring the coupling efficiency of Qβ to gH1-Qβ by densitometrically quantifying their bands following SDS-PAGE and calculating the mass contribution of each protein from their molecular weights. The coupling efficiency was found to be 0.25, so that one of four Qβ-molecules is covalently linked to a gH1 molecule. From this ratio and given the molecular weights of 14.1 kDa for Qβ and 31.3 kDa for gH1 we can attribute 37% of the total protein mass of the gH1-Qβ virus-like particle to gH1. Similarly for Panvax, given the molecular weight of the globular domain in respect to the full length HA (31.3 and 63.2 kDa respectively), we estimate that a 1.5 µg dose of Panvax contains approximately 0.7 µg gH1. As they are derived from the same particles, coupling efficiency and therefore gH1 content of gH1-Qβ(pGlu) and gH1-Qβ are equivalent. Blood samples were collected 2 weeks after the second immunization for serology studies, and spleen samples taken 1 week following the second immunization for assessment of the cellular immune response. For lung viral titer studies mice were challenged with 10^5^ plaque forming units (pfu) of pH1N1.

### Determination of Mouse Antibodies by Hemagglutination Inhibition (HAI) Test

The HAI test was performed in duplicate on individual sera taken 2 weeks after the second immunization. Two-fold serially diluted samples (25 µl per well) were incubated with 25 µl pH1N1 (8 Hemagglutinating Units) for 30 minutes at room temperature. Following the incubation, 50 µl of 1% Guinea pig RBC was added to each well, mixed, covered and then allowed to incubate for 1 hour at room temperature, after which HAI titers were recorded by visual inspection. The titer is defined as the serum dilution in which the last complete agglutination occurs.

### Determination of Mouse Antibodies by Micro-neutralization Assay

MDCK cells (ATCC) were plated at 24,000 cells per well in 96 well flat bottom tissue culture plates (Nunc) and incubated overnight at 37°C in 5% CO_2_. Each serum sample was tested in duplicate and inactivated by heating to 56°C for 30 min. Sera were serially diluted 2-fold in viral growth medium [VGM; 486.5 ml DMEM Glutamax (Gibco), 13.5 ml Hepes (Invitrogen), 2 µg/ml TPCK trypsin (Sigma)] in 96 well round bottom plates and incubated with 100 times the 50% tissue culture infective dose (TCID_50_) of pH1N1 virus, for 3 hours at 37°C in 5% CO_2_. Subsequently, 100 µl of the virus-infected sera were transferred to MDCK plates and incubated for 2 hours at 37°C in 5% CO_2_. Medium was removed from the entire plate and washed with VGM and incubated at 37°C in 5% CO_2_ for 2 days. The presence of influenza A virus was determined by ELISA to detect MDCK cell surface expressed HA. To calculate the micro-neutralization titer the average of the serum blank was used as 100% to calculate the percentage neutralization for each well. Wells with a value below 50% were considered neutralized.

### Determination of Lung Viral Titer following Challenge in Mice

Lungs were harvested, passed through a cell strainer and viral titer determined in the filtrate supernatant by calculating the TCID_50_ following incubation with MDCK cells. Each sample was assayed in quadruplicate. Briefly, MDCK cells were plated at 24,000 cells per well in 96 well flat bottom tissue culture plates (Nunc) and incubated overnight. The following day lung filtrate supernatants were serially diluted in VGM and incubated with the MDCK cells at 37°C in 5% CO_2_ for 2 hours. Following incubation, cells were washed with VGM and incubated for a further 4 days at 37°C in 5% CO_2_. The presence of influenza A virus was determined by ELISA to detect for MDCK cell surface expressed HA. Lung viral titer is expressed as Log TCID_50_ and calculated by the Reed-Meunch method [24].

### Measurement of Hemagglutinin-specific Antibody Isotype Subclasses by ELISA

Flat bottom 96 well plates (Nunc) were coated with 1 µg/ml gH1 overnight at 4°C and then blocked with 5% BSA/5% FCS in PBS for 2 hours at room temperature. Serum samples were serially diluted from 1∶50 to 1∶109350 in 1% BSA/1% FCS in PBS and transferred to the HA-coated plates. Levels of IgG1 and IgG2a specific for gH1 were measured with biotin-conjugated anti-mouse IgG1 and IgG2a, respectively (Jackson) and biotin detected with HRP-labeled streptavidin. The presence of HRP was visualized as described for the micro-neutralization assay. Antibody titers, used to calculate the IgG2a:IgG1 ratio, were defined as those dilutions that reached half the maximal OD observed for the assay. One way analysis of variance (ANOVA) on the log transformed titers was used to compare groups and found to be significant at the 5% level. This was followed up with Tukey’s multiple comparison test used (within the ANOVA model) to compare between 2 groups.

### Analysis of Vaccine-specific T cell Repertoires with Overlapping Peptides and Recombinant gH1

Overlapping peptides covering the entire sequence of gH1 and Qβ (15-mers, with a 4-aa lag) were purchased from Sigma (Sigma). Peptide sequences and composition of peptide pools for gH1 and Qβ are described in Table S1 and Table S2 respectively. Mouse splenocytes were labeled with 1 mM CFSE (Invitrogen), and were cultured in complete culture medium [cRPMI: RPMI 1640 medium (Gibco BRL), 15 mM HEPES (Gibco), 1% Non-essential amino acid (Gibco), 1 mM Sodium Pyruvate (Gibco) 1% Penicillin/Streptomycin (Gibco), 2 mM L-glutamine (Gibco), 50 µM ß2-mercaptoethanol (Sigma) and 10% heat-inactivated FCS (Hyclone)] (200,000 cells per well) with clusters of peptides (5–6 peptides/cluster, 10 nM for each peptide). Secreted cytokine levels were measured with bead-based cytokine multiplex analysis (Millipore) performed on cell culture supernatants on day 4. Also on day 4, cells were stained with 7AAD viability solution (eBiosciences), and labeled with CD8-PECy7 (eBiosciences), CD3-eFluor450 (eBiosciences), CD45-PacificOrange (Invitrogen) and CD4-Qdot605 (Invitrogen) before analysis of T cell proliferation by CFSE dilution visualized by flow cytometry using a LSRII (BD).

The frequencies of CD4^+^ and CD8^+^ T-cells producing the cytokines IFNγ, IL-4 and IL-13 were measured following stimulation of splenocytes with gH1 or Qβ peptide clusters, or recombinant gH1 (20 µg/ml), in the presence of anti-CD28 (1 µg/ml) (BD). After 2 hours of stimulation, Brefeldin A (10 µg/ml) was added and incubated for an additional 10 hours. Cells were washed, fixed and permeabilized with the Cytofix/Cytoperm kit (BD), washed with Permwash and labeled with the following monoclonal antibodies: CD3-PECy5 (BD), IL4-PE (eBiosciences), CD4-APC (eBiosciences), CD8-APCCy7 (BioLegend), IFNγ-PECy7 (eBiosciences) and IL13-A488 (eBiosciences). Labeled cells were analyzed by flow cytometry using a Macsquant Analyzer (Miltenyi).

### Ferret Immunizations and Antibody Neutralization Assay

Male and female ferrets, aged 4 to 6 months old, were obtained from Marshall Farms (North Rose, NY). Ferrets were free of antibodies against circulating H1N1 and H3N2 strains, as tested by ELISA using infected cells as antigen as previously described [25]. Ferrets were immunized twice intramuscularly on days 0 and 21 with 100 µg non-adjuvanted gH1-Qβ (6 ferrets), 100 µg alum-adjuvanted gH1-Qβ (6 ferrets) or 15 µg licensed pH1N1 vaccine Panvax (4 ferrets). Control animals received PBS in place of the vaccine (4 ferrets). Serum samples were collected on day 21 prior to the second immunization and before the challenge on day 42.

Neutralizing antibody titers against pH1N1 were quantified by micro-neutralization assay following a modified WHO protocol. Briefly, starting at a 1∶10 dilution, two fold serum dilutions were performed in quadruplicate in a 96-well plate, mixed with 100 TCID_50_ units and incubated for 20 minutes at room temperature, after which MDCK cells were added to each well. The plates were incubated for 2 days at 37°C in 5% CO_2_, washed once with PBS (Invitrogen) diluted in two volumes of ddH_2_O, dried and heat-fixed over night at 65°C. Infected cells were detected by immuno-staining using a ferret antiserum against pH1N1, and a peroxidase-labeled antibody against ferret IgG. Neutralizing antibody titers are expressed as the reciprocal value of the last dilution at which no infected cells were detected.

### Ferret Challenge

On Day 42, all animals were challenged with 10^5^ TCID_50_ of H1N1 A/California/07/09. Rectal temperature and body weight were recorded daily, alongside nasal wash fluid collection, from days 42 to 48. Activity and respiratory clinical signs were scored daily using a 0–1–2 scoring system with 0 indicating no changes, 1 indicating reduced activity and mild respiratory signs, and 2 representing depressed behavior and moderate respiratory signs. The viral load in nasal wash fluids was determined by limited dilution method and expressed as 50% tissue culture infectious doses (TCID_50_) per ml.

### Ethics Statement

Mouse studies were approved by the Singapore DSO Animal Care and Use Committee (DSO-ACUC #09-81) and the Singapore Agency for Science, Technology and Research Institutional Animal Care and Use Committee (A-STAR-IACUC #100539).

Ferret experiments were approved by the Institutional Care and Use Committee of the INRS (Institute Armand-Frappier) protocol # 0911-02.

## Results

### Alum-adjuvanted gH1-Qβ Performs as well as Panvax in Mice Vaccinated and then Challenged with the 2009 Pandemic Influenza Strain

The globular domain of A/California/07/2009 strain (pH1N1) was expressed in *E. coli*, purified as insoluble inclusion bodies, and refolded into its native form (for details see accompanying paper (A. Jegerlehner, F. Zabel, A. Langer, K. Dietmeier, G. T. Jennings, P. Saudan and M. F. Bachmann, submitted for publication)).

This recombinant HA (gH1) was used to produce the conjugate vaccine gH1-Qβ by covalently linking it to the surface of recombinant *E.coli*-produced Qβ VLPs derived from the coat protein of bacteriophage Qβ, as described in the accompanying paper (A. Jegerlehner, F. Zabel, A. Langer, K. Dietmeier, G. T. Jennings, P. Saudan and M. F. Bachmann, submitted for publication).

**Figure 1:**
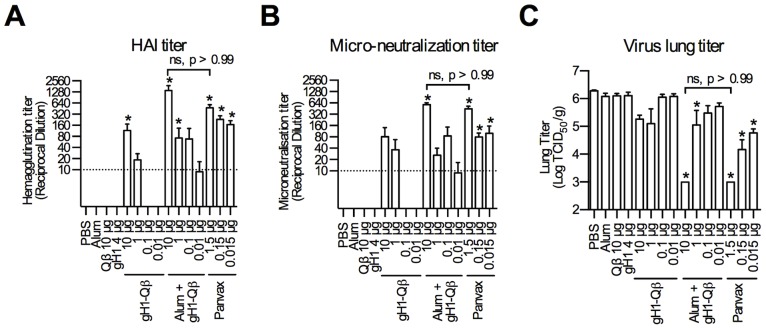
Alum-adjuvanted gH1-Qβ performs as well as Panvax in mice vaccinated and then challenged with the 2009 pandemic influenza strain. Groups of five BALB/c mice were immunized subcutaneously at days 0 and 28 with indicated doses of gH1-Qβ (either alone or adjuvanted with alum) or Panvax. Shown are the means (and standard errors) of serum hemagglutination inhibition (HAI) titers against pH1N1 2 weeks post second dose (A), virus micro-neutralization titers against pH1N1 2 weeks post second dose (B) and virus lung titers following challenge with pH1N1 3 weeks post second dose (C). One way ANOVA on the log transformed titers was used to compare groups and the significance level set at 5%. All ANOVA tests were found to be significant and were followed up with Tukey’s honest significant difference test to compare between any 2 groups. Significant differences between vaccine response and PBS control are outlined with *, p<0.05. Reported p values are adjusted to account for multiple comparisons. The abbreviation, ns, indicates no significant difference between the indicated groups.

To evaluate the performance of this Qβ-based vaccine relative to the traditionally-produced Panvax pH1N1 vaccine, BALB/c mice were immunized at days 0 and 28 with the vaccine formulations, and serum samples taken for measurement of HAI and neutralizing antibody titers to pH1N1 2 weeks after the second dose (Figure 1A & B). At a dose of 10 µg, alum-adjuvanted gH1-Qβ induced comparable HAI and neutralization titres to a 1.5 µg dose of Panvax. For the purpose of directly comparing the dose of the two vaccines it needs to be clarified that a 10 µg dose of alum-adjuvanted gH1-Qβ has a gH1 content of 3.7 µg and a 1.5 µg dose of Panvax has a gH1 content of approximately 0.7 µg. Following challenge with influenza virus, three-log lower viral titers were observed in the lungs of mice immunized with alum-adjuvanted gH1-Qβ, again matching the performance of Panvax (Figure 1C). Alum was required for this reduction in viral titer, as non-adjuvanted gH1-Qβ, even at the maximum dose, did not provide sufficient control of the virus in the lung (Figure 1C). Significantly greater HAI titers were also observed for gH1-Qβ over uncoupled gH1 (p<0.01), demonstrating the importance of the VLP component of the vaccine. When comparing alum-adjuvanted gH1-Qβ and Panvax at the more similar doses of 1 µg (0.37 µg gH1 content) and 1.5 µg (0.7 µg gH1 content) respectively, Panvax demonstrated higher HAI titers (p<0.01), higher neutralization titres (p<0.01) and lower viral titers in the lungs of mice (p<0.0001). In summary, immunization of mice with 10 µg of adjuvanted gH1-Qβ (3.7 µg globular HA content) compared favorably with 1.5 µg of unadjuvanted Panvax (0.7 µg globular HA content) in terms of HAI and neutralizing antibody titers, and achieved control of influenza in the lung following pH1N1 challenge.

### Alum-adjuvanted gH1-Qβ Enhances the Breadth of the T cell Response against HA and Promotes Promotes IFNγ Production

**Figure 2:**
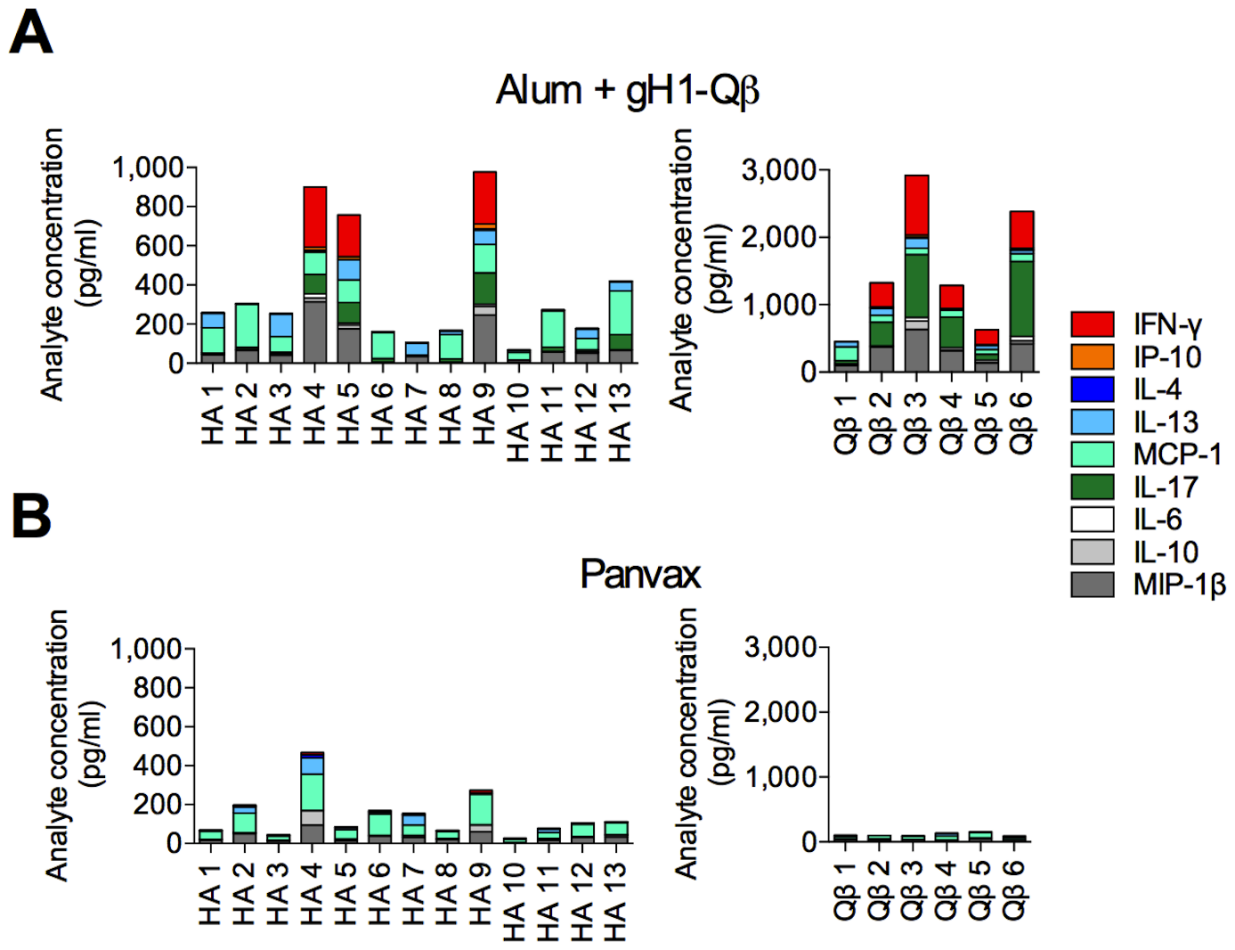
Alum-adjuvanted gH1-Qβ induces broad gH1 and Qβ specific T cell responses characterized by the production of IFNγ. HA and Qβ specific T cell responses were measured in pooled splenocytes from three BALB/c mice by secreted cytokine concentration after stimulation with peptide pools spanning the HA and Qβ regions of the gH1-Qβ vaccine. Mice were immunized with 10 µg alum-adjuvanted gH1-Qβ and 1.5 µg of Panvax. Each bar represents the total cytokine response to each peptide pool and each box the response to each cytokine. Names designated to each peptide pool are indicated on the x axes. Each data point is the response from splenocytes pooled from three mice.

As our novel vaccination strategy also aimed to induce T cell responses to influenza, we employed EPIMAX (EPItope MAXimum), an approach normally reserved for human studies [26]–[28], to identify the regions of both HA and Qβ that were recognized by cytokine-secreting T cells from vaccinated mice. Peptide libraries spanning the length of gH1 and bacteriophage Qβ coat protein (Qβ) were generated, and pools of these peptides (5 peptides per pool) used to stimulate 5,6 carboxyfluorescein diacetate succinimidyl ester (CFSE)-labeled splenocytes from vaccinated mice. We then measured T cell proliferation and the amount of a range of cytokines in the cell culture supernatant 4 days after peptide re-stimulation (Figure 2 & Figure S1). Splenocytes from mice immunized with alum-adjuvanted gH1-Qβ typically produced 2–3 times more total cytokines to any given HA peptide pool compared to cells from mice immunized with Panvax. The Qβ component of the vaccine was also highly immunogenic. HA-specific responses from gH1-Qβ vaccinated animals were dominated by the production of IFNγ, while this response was lacking in cells from animals receiving Panvax. Interestingly, our novel vaccine induced T cells that responded to peptide pools which were largely non-immunogenic in mice immunized with Panvax. Taken together, the EPIMAX technique identified regions of the gH1-Qβ vaccine containing T cell epitopes, and determined the quality and magnitude of the T cell response to those epitopes. The gH1- Qβ vaccine induces a greater breadth and strength of T cell cytokine response than Panvax, including the production of substantial amounts of IFN- γ, which is known to drive a desirable type 1 T cell response.

### The Presence of ssRNA in gH1-Qβ VLPs is Responsible for an Increased IgG2a bias in the Influenza-specific Antibody Repertoire

**Figure 3:**
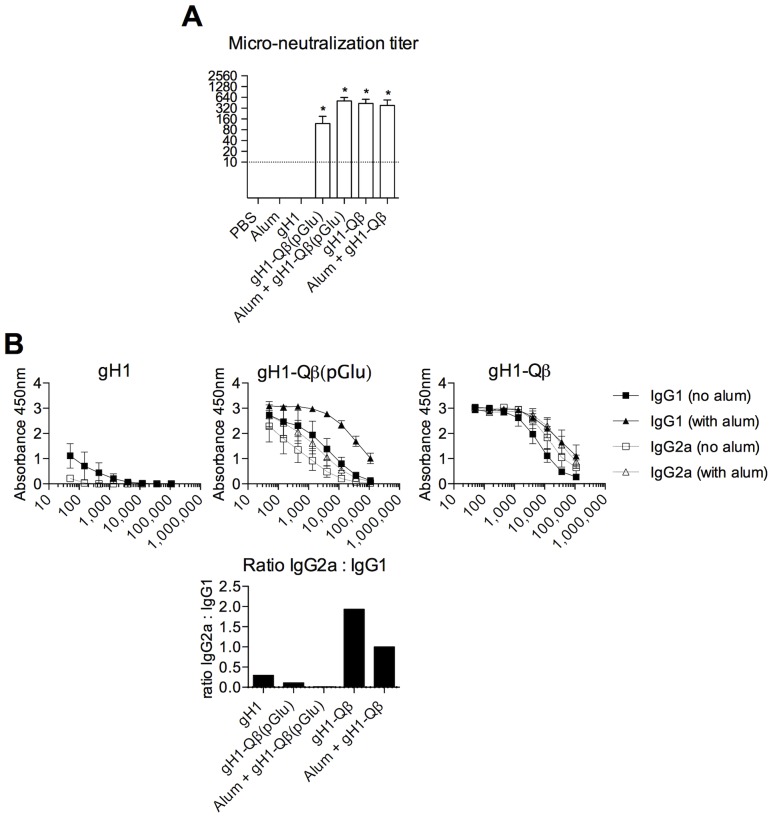
The presence of ssRNA in gH1-Qβ VLPs is responsible for an increased IgG2a bias in the influenza-specific antibody repertoire. Groups of five BALB/c mice were immunized subcutaneously at days 0 and 28 with 4 µg gH1, 10 µg gH1-Qβ(pGlu) or 10 µg gH1-Qβ, either alone or adjuvanted with Alum. Antibody responses were assayed 2 weeks post second dose. (A) Mean (and standard error) of virus neutralization titers against pH1N1. One way ANOVA on the log transformed titers was used to compare groups and the significance level set at 5%. The ANOVA test was significant and Tukey’s honest significant difference test was then used to compare between any 2 groups. Significant differences between vaccine response and PBS control are outlined with *, p<0.05. (B) Mean (and standard error) of gH1-specific IgG1 and IgG2a antibody isotype responses determined by ELISA. Responses are also shown as ratios of gH1-specific IgG2a:IgG1 isotype geometric mean titers, with titers defined as those dilutions that reached half the maximal OD observed for the assay.

The ssRNA naturally packaged into the VLP during expression in *E.coli* is an agonist of TLR7 and has been shown to induce Th1 responses and antibody isotype switching towards IgG2a [29], [30]. We therefore asked what the combined effect of alum and ssRNA would be on humoral immunity in our murine immunization model. To test the influence of ssRNA on the antibody response to gH1-Qβ we prepared VLPs without packaged RNA by disassembling them and reassembling in the presence of the polyanionic molecule polyglutamic acid, as previously described [31], [32]. The resulting particles, Qβ(pGlu), were conjugated to gH1 to give the vaccine gH1- Qβ(pGlu). Mice were immunized with unconjugated gH1(no Qβ), gH1- Qβ(pGlu) or gH1- Qβ, both with and without alum, and the resulting antibody response measured (Figure 3). While gH1 alone failed to induce detectable antibody responses, either gH1- Qβ(pGlu) or gH1- Qβ were enough to stimulate similarly high titers of neutralizing antibodies in immunized mice (Figure 3A). These titers were approximately 4 fold higher for gH1-Qβ over gH1-Qb(pGlu) but this increase was not statistically significant (p<0.05). However, when ssRNA was lacking in the VLPs, the humoral response was dominated by IgG1 (Figure 3B and Figure S2), while in the presence of ssRNA there was a significant increase in the production of the more desirable IgG2a antibody subclass (p<0.01).

### The Presence of ssRNA in gH1-Qβ VLPs is Responsible for an Increased Th1 Bias in the Influenza-specific CD4+ T cell Response

**Figure 4:**
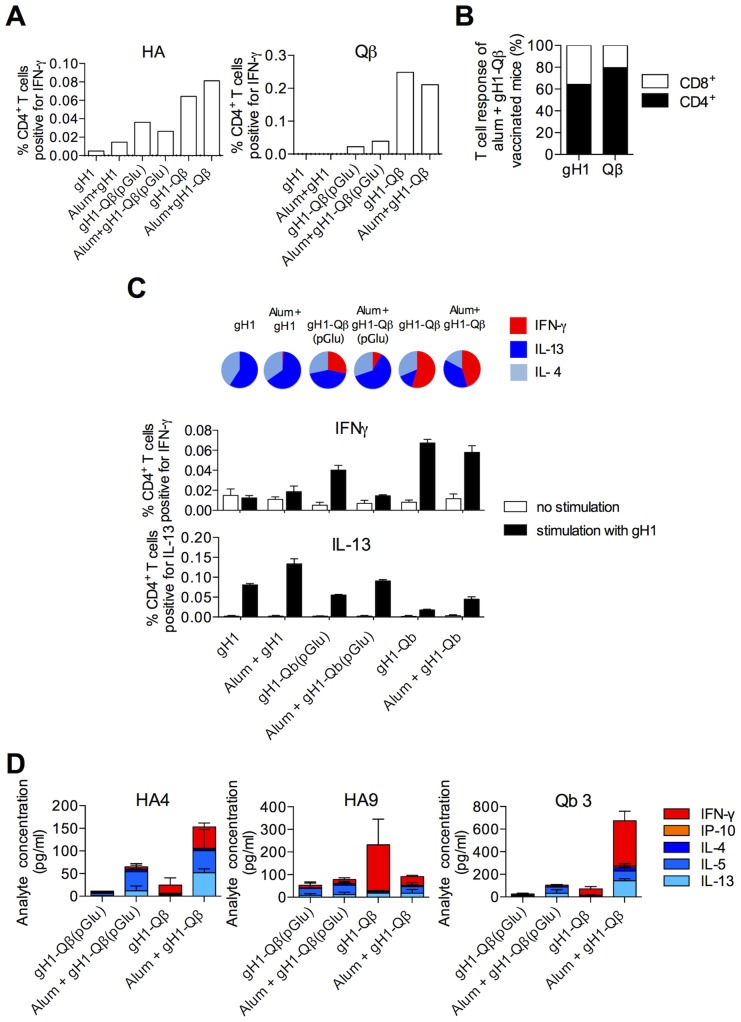
The presence of ssRNA in Qβ VLPs is responsible for an increased Th1 bias in the influenza-specific CD4+ T cell response. Mice were immunized with 1 µg gH1, 10 µg gH1-Qβ or 10 µg gH1-Qβ(pGlu) as indicated, either non-adjuvanted or adjuvanted with alum, and T cell responses measured two weeks post second dose. (A) Frequency of HA and Qβ specific IFNγ-producing CD4^+^ T cells was determined by intracellular staining after stimulation with peptides from the HA (peptide pools HA 4, HA5, HA6, HA8 & HA9) and Qβ (peptide pools Qβ 2, Qβ 3, Qβ 4 & Qβ 6) regions of the gH1-Qβ vaccine. Each data point is the total response obtained by adding together individual responses measured for each peptide pool. Mouse splenocytes were pooled from at least three mice and data are representative of at least two experiments performed with similar results. Unstimulated samples were subtracted from the peptide-stimulated samples. (B) Plot of the relative proportion of CD4^+^ and CD8^+^ contributions to IFNγ^+^ T cell responses, as obtained in (A), to ex-vivo stimulation of HA and Qβ peptides for mice immunized with 10 µg alum-adjuvanted gH1-Qβ. (C) Histogram showing the frequency of CD4^+^ T cells, determined by intracellular labeling, that are positive for the indicated cytokine following ex-vivo stimulation with recombinant gH1 or diluent alone. Pie-charts display responses plotted as relative frequency of IFNγ, IL-4 and IL-13 producing CD4^+^ T cells, with values from unstimulated samples subtracted from the Ag-stimulated sample. Each data point is the mean response of three replicate measurements from splenocytes pooled from at least three mice and is representative of at least two experiments performed with similar results. (D) Total secreted cytokine levels to the indicated HA and Qβ peptide pools. Each data point is the response from splenocytes pooled from at least three mice and is the mean of two independent experiments (error bars represent the range).

Having established the roles played by alum, Qβ and ssRNA in the antibody response to our vaccine, we then extended the study to include a detailed characterization of their contribution to the T cell immunity we had observed (Figure 2). Mice were immunized with unconjugated gH1(no Qβ), gH1-Qβ(pGlu) or gH1-Qβ and T-cell cytokine responses were then measured following ex-vivo stimulation of splenocytes with HA and Qβ peptide pools. Intracellular labeling for IFNγ confirmed that mice immunized with alum-adjuvanted gH1-Qβ responded to both gH1 and Qβ components of the vaccine (Figure 4A). The frequency of IFNγ-producing T cells was markedly higher when animals had been vaccinated with VLPs containing ssRNA than when ssRNA was absent, indicating their requirement for TLR7 stimulation. Interestingly the alum-adjuvanted gH1-Qβ also induced some gH1- and Qβ -specific IFNγ production from CD8+ T cells, though the response remained dominated by CD4+ lymphocytes (64% CD4^+^ versus 36% CD8^+^ for HA peptides and 80% CD4^+^ versus 20% CD8^+^ for Qβ peptides) (Figure 4B).

We then addressed the question of T cell polarization in the presence of the ssRNA and alum adjuvants. As expected, immunization with Th2-promoting alum in the absence of ssRNA (alum+gH1-Qβ(pGlu)) led to a response dominated by CD4^+^ T cells producing the Th2 cytokines IL-4 and IL-13 following ex-vivo restimulation with gH1 (Figure 4C). However, the inclusion of ssRNA in the VLP vaccine was able to drive IFNγ production in a markedly higher proportion of CD4+ T cells, even in the presence of alum.

Assaying the levels of cytokines produced at Day 4 confirmed the induction of IFNγ in response to the most immunodominant gH1 and Qβ peptide pools and the requirement for the TLR7-stimulatory ssRNA (Figure 4D). Again the inclusion of ssRNA within the VLP was able to moderate the Th2-promoting effect of alum and induce substantial vaccine-specific IFNγ-production.

### In a Ferret Challenge Model gH1-Qβ Immunization Decreases Morbidity and Reduces Viral Titer in Nasal Lavage

Ferrets are widely used to predict influenza vaccine efficacy in humans [33], [34]. We immunized ferrets with 100 µg doses of gH1-Qβ both with and without alum adjuvant and compared immunogenicity and efficacy to a 15 µg dose of the licensed pH1N1 vaccine, Panvax. As the mouse dose ranging studies described above (Figure 1) found comparable responses between a 10 µg dose of alum-adjuvanted gH1-Qβ and a 1.5 µg dose of Panvax, and considering that the adult human dose of Panvax at 15 µg is ten fold greater than this, we projected a human dose for gH1-Qβ of 100 µg for investigation in ferrets. The globular HA content of a 100 µg dose of gH1- Qβ and 15 µg dose of Panvax are 37 µg and 7 µg respectively.

**Figure 5:**
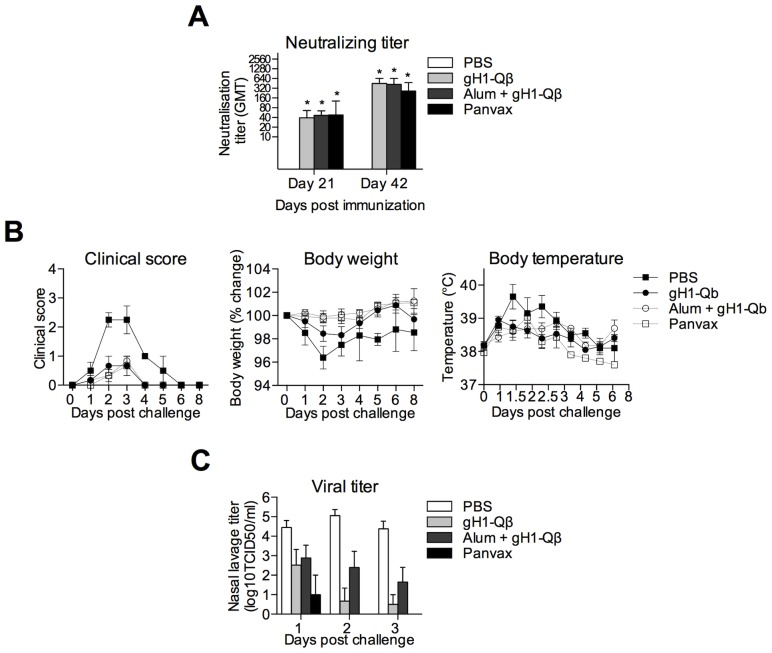
Alum-adjuvanted gH1-Qβ elicits protective immunity in Ferrets. Ferrets were immunized twice intramuscularly on days 0 and 21 with 100 µg of non-adjuvanted gH1-Qβ, 100 µg alum-adjuvanted gH1-Qβ or 15 µg Panvax. (A) Geometric mean (and 95% confidence intervals) for neutralizing antibody responses. A two-way repeated-measures ANOVA was performed on the log transformed titers with time point and vaccine as the factors. Both factors and their interaction were found to be significant at the 5% significance level and Bonferoni’s corrected multiple comparison test used to compare between 2 groups. Significant differences between vaccine response and PBS control are outlined with *, p<0.05. (B) Mean (and standard error) of activity and respiratory scores (0 indicating no changes, 1 indicating reduced activity and mild respiratory signs, and 2 representing depressed behavior and moderate respiratory signs), body weight and body temperature measured over the 6 days following challenge with pH1N1. Clinical scores and body weight for both the alum-adjuvanted and non-adjuvanted gH1-Qβ and Panvax were not significant from each other (p>0.05), but significantly different from the control PBS group as determined by linear mixed model (p<0.05). (C) Mean (and standard error) of viral titers in nasal washes at indicated days after challenge with pH1N1. Titers for all vaccines were significantly different from the control PBS group as determined by linear mixed model (p<0.01).

After a single immunization all three vaccines induced significant (p<0.05 compared to PBS control) and similar neutralizing antibody titers, which increased approximately ten-fold following administration of a second dose (Figure 5A). As expected from the relatively mild pH1N1 strain, infection did not cause severe disease in the ferrets, although clear symptoms of influenza were present in the control, PBS-vaccinated, animals. Immunization with gH1-Qβ in the presence or absence of alum conferred significant protection against clinical symptoms of disease (p<0.05) and significantly reduced the weight loss associated with infection (p<0.05)(Figure 5B), as did Panvax.

In addition, while high titers of virus were detected in the nasal washes of the PBS control animals on days 1, 2 and 3 post challenge, the vaccinated groups had at least ten-fold lower virus concentrations at these time points (p<0.01) (Figure 5C), clearly demonstrating the ability of our novel VLP-based vaccine to control infection *in vivo* in a widely-accepted model of the human disease. Between the vaccinated groups there was no significant difference in viral titers detected between unadjuvanted gH1-Qβ and Panvax, and between unadjuvnated gH1-Qβ and alum-adjuvanted gH1-Qβ. There was a significant difference in viral titers in nasal washes detected between unadjuvanted gH1-Qβ and Panvax (p<0.05).

## Discussion

The 2009 H1N1 pandemic highlighted the need for an improved influenza response both in terms of shortening the time required to produce the vaccine and increasing capacity to make sufficient quantities [2]. Production of HA in *E. coli* can address both these problems. DNA constructs incorporating the HA sequence of a newly emerging pandemic influenza strain can be prepared in 1–2 weeks and be produced in rapidly growing *E. coli* as a high proportion of the total microbial biomass [35].

In this study we assessed a novel pH1N1 vaccine produced by coupling the *E. coli*-produced HA globular domain (gH1) to VLPs of the bacteriophage Qβ. We hypothesized that conjugation of gH1 to Qβ would enhance its immunogenicity and efficacy through a number of mechanisms. Firstly, presentation of gH1 on the surface of viral-like-particles in a repetitive fashion is a strong activation signal for B cells, facilitating the cross-linking of B cell receptors [36]–[38]. Secondly, the particulate nature of VLPs makes them likely to be more efficiently taken up by APCs, facilitating the presentation of gH1-specific and Qβ-specific epitopes on MHC molecules for T cell priming [22]. Lastly, as mentioned earlier, the bacterial RNA that is incorporated into the VLP during recombinant expression is a TLR7 ligand that can activate APCs and B cells upon uptake of the VLP [22]. Alongside the efficacy of VLP-based strategies, they are known to be safe in humans and have already been used as a carrier coupled to five different antigens. These antigens (and indications) include nicotine (smoking cessation), angiotensin II (hypertension), amyloid-β (Alzheimer’s disease), IgE (allergy) and a synthetic 16-amino-acid sequence of the allergen Der p 1 [39]–[44]. Testing of these VLP-based vaccines has proven safe in seventeen clinical studies (phases 1 and 2) with more than 1000 subjects in doses up to 900 µg [39]–[44].

When used to immunize mice gH1-Qβ induced significantly higher HAI and neutralizing antibody titers than the unconjugated recombinantly-produced HA (gH1). When administered with alum, a 10 µg dose of gH1-Qβ (3.7 µg globular HA content) induced serum antibodies in mice with HAI and neutralizing titers as high as a 1.5 µg dose of the licensed pH1N1 vaccine Panvax (0.7 µg globular HA content), and significantly reduced viral titers in the murine lung following challenge with pH1N1. These findings were observed at a 10 µg dose of gH1-Qβ and used to establish a projected human dose of 100 µg. The addition of alum to the gH1-Qβ vaccine was required for this reduction in lung viral titer, and was important, especially at lower vaccine doses, for the development of influenza-specific antibodies.

We also showed that while Panvax failed to induce substantial T cell cytokine responses to HA peptides, our novel vaccine not only induced prolific responses, but increased the repertoire of peptides recognized by the murine T cell pool. This observation is important as the increased breath of response makes it less likely that antigenic drift will allow a virus to evade the T cell response. Although the vaccine will be designed to match the HA sequence of an emerging pandemic strain there remains the possibility that genetic variation in this strain during the course of the pandemic will result in antigenic changes. This antigenic drift was not observed with the 2009 H1N1 pandemic strain despite genetic variation in HA [45], [46]. However, the performance of the vaccine in the face of potential antigenic changes is an important focus of future studies. Extensive mapping of the magnitude and quality of this T cell response to the vaccine revealed that delivery of gH1 conjugated to Qβ VLPs induced more of a type 1 T cell response, evidenced by increased production of IFNγ compared to Panvax. The response was driven by CD4^+^ T cells specific for both HA and Qβ. We speculate that this CD4^+^ T-cell response induced by the alum-adjuvanted gH1-Qβ vaccine likely provides help for HA-specific B-cells in mounting the antibody response, and could be another factor that helps explain how the bacterially produced vaccine based solely on the globular domain of HA can reach comparable efficacy to that of the full length native HA antigen. Furthermore we cannot rule out the possibility that in addition to their helper function, CD4^+^ T cells induced by the vaccine may have a direct effector function against subsequent influenza infection. Studies have shown that influenza-specific memory CD4^+^ T cells can directly protect against influenza infection in the absence of B cells or CD8^+^ T cells [47], [48]. This direct control of influenza infection is dependent on IFNγ, and it thought to be mediated through the induction of an antiviral state in the lung combined with the infiltration of innate immune cells [47], [48]. In addition effector CD4^+^ T cells have been shown utilize a perforin-dependent cytolytic mechanism to fight influenza infection and control influenza titers in a perforin-dependent manner [48]. The contribution of these CD4^+^ T cell mediated anti-viral activities in directly controlling influenza infection following vaccination with the gH1-Qβ vaccine require further studies for investigation.

The ssRNA contained within the VLP of the vaccine had the potential to act both as an adjuvant and an immune-modulator, through its known Th1-promoting activity. However, alum, known for its ability to induce robust antibody responses in many vaccines, is a Th2-polarizing adjuvant. Therefore we wished to understand the interplay between these two components of the vaccine formulation in order to achieve optimal induction of anti-influenza immunity. We first produced VLPs lacking ssRNA for comparison with the complete gH1-Qβ vaccine, both with and without alum, and then compared both humoral and cellular immune responses to the vaccines. We found that the ssRNA component of the VLP-based vaccine was responsible for both a Th1-type cytokine response, and inducing an antibody response dominated by IgG2a. These effects persisted in the presence of alum, under conditions which we showed could induce protection during infection of mice. This indicates that by employing the joint adjuvant strategy of ssRNA and alum, we have designed an immunization regimen that can induce high titers of protective antibody, with a desirable IgG2a subclass dominance and a potent IFNγ response from CD4^+^ T cells. This could represent an exciting development based on *in vitro* studies that established that IFNγ has an anti-viral role against human influenza [49], and IgG2a is more effective than IgG1 in preventing intracellular virus replication [50]. T cell responses also correlate with disease protection against influenza; increased frequency of CD4^+^ T cells in particular correlate with lower virus shedding and less severe illness following infection with H3N2 or H1N1 viruses in seronegative humans [11]. Furthermore T cell responses are better correlates of vaccine protection in the elderly, with the ratio of IFNγ to the immunosuppressive cytokine IL10 better correlating with protection against influenza than serum antibody titers [12].

As seen in mice, in ferrets the performance of the VLP vaccine was as good as the licensed pH1N1 vaccine, Panvax, in terms of inducing neutralizing antibodies following immunization and reducing the severity of clinical disease. Our novel vaccine was able to induce marked reductions in influenza titer in nasal lavage from as early as 1 day post infection, which could be important in the pandemic setting to reduce transmission rates through the population. Although the neutralizing antibody titers and clinical scores were comparable a significantly lower influenza titer in ferret nasal lavages was observed in the Panvax group compared to the alum-adjuvanted gH1-Qβ group. This was not observed in mice where lung viral titers were comparable between these two groups and the alum-adjuvanted gH1-Qβ group clearly outperformed the unadjuvanted gH1-Qβ group. A lack of adjuvant effect with alum in ferrets has been has been previously observed in pandemic influenza vaccines [51], although similar studies have also demonstrated a benefit from use of the adjuvant [52]. Alum is generally not used as adjuvant in seasonal influenza vaccines as clinical studies have revealed little benefit [53]. Studies in man with subvirion H5N1 vaccines have also revealed little benefit from the adjuvant, with alum failing to enhance immunogenicity of vaccines containing low antigen content [54], [55]. To address this and understand the benefit of adjuvanting gH1-Qβ with alum adjuvant, a Phase 1 clinical assessment of the vaccine is underway both with and without alum adjuvant.

Overall, we conclude that gH1-Qβ has the potential to be the basis of a flexible, rapidly scalable pandemic vaccine technology with demonstrated immunogenicity and efficacy in both the mouse and ferret models. The high HAI and neutralizing antibody titers, which form the conventional basis for influenza vaccine development, and the potent influenza-specific Th1 response, reveal the promise of an optimal influenza vaccine. Phase 1 clinical assessment of the gH1-Qβ vaccine both with and without alum adjuvant is currently ongoing and will reveal how these encouraging pre-clinical data translate into the human setting.

## Supporting Information

Figure S1 **Alum-adjuvanted gH1-Qβ induces gH1 and Qβ specific T cell responses.** Proliferation of CD4^+^ T cells in response to the HA9 and Qβ3 peptide pools. CFSE-labeled PBMCs from mice immunized with (A) 10 µg of alum-adjuvanted gH1-Qβ or (B) PBS alone (placebo), were cultured with the indicated peptide pools and CFSE dilution analyzed at day 4. Percentages of proliferating cells in the gate region are indicated.
(TIF)

Figure S2 **Antigen-specific IgG1 and IgG2a antibody isotype titers were determined by ELISA against gH1.** Titers are defined as those dilutions that reached half the maximal OD observed for the assay. Geometric means and 95% confidence intervals are shown. Groups of five BALB/c mice were immunized subcutaneously at days 0 and 28 with 4 µg gH1, 10 µg gH1-Qβ(pGlu) or 10 µg gH1-Qβ, either alone or adjuvanted with Alum, or with 1.5 µg of Panvax. Antibody responses were assayed 2 weeks post second dose. One way ANOVA on the log transformed titers was used to compare groups and the significance level set at 5%. The ANOVA test was significant and Tukey’s honest significant difference test was then used to compare between any 2 groups (*, p<0.01).
(TIF)

Table S1 **Sequences of HA (A/California/07/2009) specific peptides used for re-stimulation of splenocytes from vaccinated mice.**
(DOCX)

Table S2 **Sequences of Qβ specific peptides used for re-stimulation of splenocytes from vaccinated mice.**
(DOCX)
