# Supplementary figures and images for: Enhanced Neutralizing Antibody Titers and Th1 Polarization from a Novel Escherichia coli Derived Pandemic Influenza Vaccine
Source: PLoS One. 2013 Oct 18;8(10):e76571. doi: 10.1371/journal.pone.0076571 (PMC3799843; doi:10.1371/journal.pone.0076571)

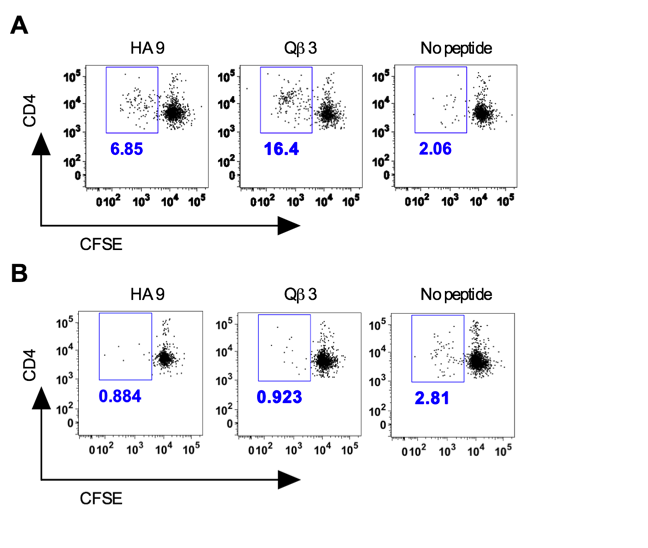

Supplement: Figure S1 — Alum-adjuvanted gH1-Qβ induces gH1 and Qβ specific T cell responses. Proliferation of CD4+ T cells in response to the HA9 and Qβ3 peptide pools. CFSE-labeled PBMCs from mice immunized with (A) 10 µg of alum-adjuvanted gH1-Qβ or (B) PBS alone (placebo), were cultured with the indicated peptide pools and CFSE dilution analyzed at day 4. Percentages of proliferating cells in the gate region are indicated. (TIF) [file pone.0076571.s001.tif]

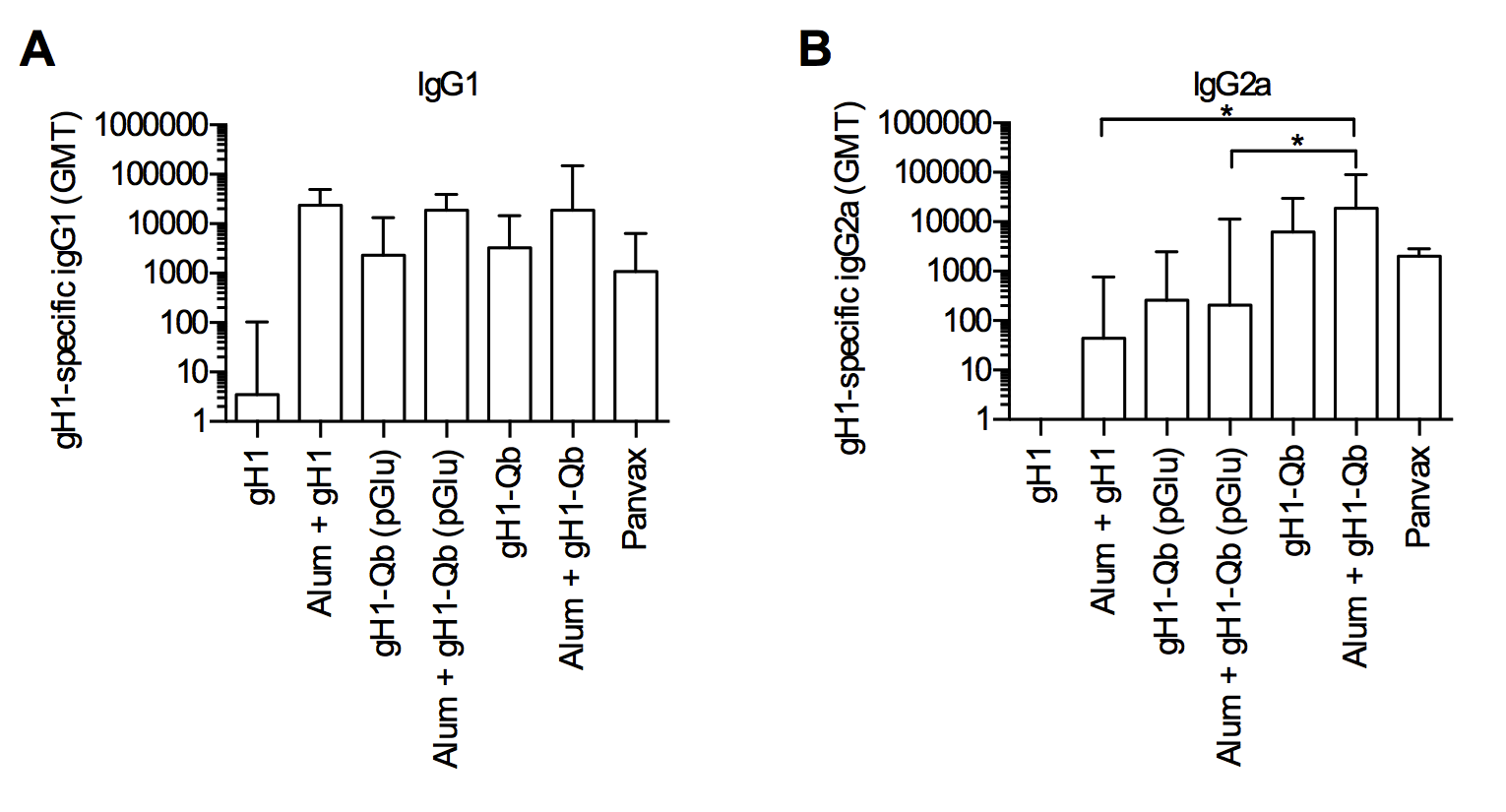

Supplement: Figure S2 — Antigen-specific IgG1 and IgG2a antibody isotype titers were determined by ELISA against gH1. Titers are defined as those dilutions that reached half the maximal OD observed for the assay. Geometric means and 95% confidence intervals are shown. Groups of five BALB/c mice were immunized subcutaneously at days 0 and 28 with 4 µg gH1, 10 µg gH1-Qβ(pGlu) or 10 µg gH1-Qβ, either alone or adjuvanted with Alum, or with 1.5 µg of Panvax. Antibody responses were assayed 2 weeks post second dose. One way ANOVA on the log transformed titers was used to compare groups and the significance level set at 5%. The ANOVA test was significant and Tukey’s honest significant difference test was then used to compare between any 2 groups (*, p<0.01). (TIF) [file pone.0076571.s002.tif]
