# Supplementary material for: Enhanced Neutralizing Antibody Titers and Th1 Polarization from a Novel Escherichia coli Derived Pandemic Influenza Vaccine
Source: PLoS One. 2013 Oct 18;8(10):e76571. doi: 10.1371/journal.pone.0076571 (PMC3799843; doi:10.1371/journal.pone.0076571)
Supplement: Table S1 — Sequences of HA (A/California/07/2009) specific peptides used for re-stimulation of splenocytes from vaccinated mice. (DOCX) [file pone.0076571.s003.docx]

**TABLE S1 Sequences of HA (A/California/07/2009) specific peptides used for re-stimulation of splenocytes from vaccinated mice.**

| **Protein** | **Peptide #** | **Peptide Cluster** | **Amino Acid Sequence** |
| --- | --- | --- | --- |
| HA (A/California/04/2009) | 1 | HA 1 | MLLEDKHNGKLCKLR |
| HA (A/California/04/2009) | 2 | HA 1 | DKHNGKLCKLRGVAP |
| HA (A/California/04/2009) | 3 | HA 1 | GKLCKLRGVAPLHLG |
| HA (A/California/04/2009) | 4 | HA 1 | KLRGVAPLHLGKCNI |
| HA (A/California/04/2009) | 5 | HA 1 | VAPLHLGKCNIAGWI |
| HA (A/California/04/2009) | 6 | HA 2 | HLGKCNIAGWILGNP |
| HA (A/California/04/2009) | 7 | HA 2 | CNIAGWILGNPECES |
| HA (A/California/04/2009) | 8 | HA 2 | GWILGNPECESLSTA |
| HA (A/California/04/2009) | 9 | HA 2 | GNPECESLSTASSWS |
| HA (A/California/04/2009) | 10 | HA 2 | CESLSTASSWSYIVE |
| HA (A/California/04/2009) | 11 | HA 3 | STASSWSYIVETPSS |
| HA (A/California/04/2009) | 12 | HA 3 | SWSYIVETPSSDNGT |
| HA (A/California/04/2009) | 13 | HA 3 | IVETPSSDNGTCYPG |
| HA (A/California/04/2009) | 14 | HA 3 | PSSDNGTCYPGDFID |
| HA (A/California/04/2009) | 15 | HA 3 | NGTCYPGDFIDYEEL |
| HA (A/California/04/2009) | 16 | HA 4 | YPGDFIDYEELREQL |
| HA (A/California/04/2009) | 17 | HA 4 | FIDYEELREQLSSVS |
| HA (A/California/04/2009) | 18 | HA 4 | EELREQLSSVSSFER |
| HA (A/California/04/2009) | 19 | HA 4 | EQLSSVSSFERFEIF |
| HA (A/California/04/2009) | 20 | HA 4 | SVSSFERFEIFPKTS |
| HA (A/California/04/2009) | 21 | HA 5 | FERFEIFPKTSSWPN |
| HA (A/California/04/2009) | 22 | HA 5 | EIFPKTSSWPNHDSN |
| HA (A/California/04/2009) | 23 | HA 5 | KTSSWPNHDSNKGVT |
| HA (A/California/04/2009) | 24 | HA 5 | WPNHDSNKGVTAACP |
| HA (A/California/04/2009) | 25 | HA 5 | DSNKGVTAACPHAGA |
| HA (A/California/04/2009) | 26 | HA 6 | GVTAACPHAGAKSFY |
| HA (A/California/04/2009) | 27 | HA 6 | ACPHAGAKSFYKNLI |
| HA (A/California/04/2009) | 28 | HA 6 | AGAKSFYKNLIWLVK |
| HA (A/California/04/2009) | 29 | HA 6 | SFYKNLIWLVKKGNS |
| HA (A/California/04/2009) | 30 | HA 6 | NLIWLVKKGNSYPKL |
| HA (A/California/04/2009) | 31 | HA 7 | LVKKGNSYPKLSKSY |
| HA (A/California/04/2009) | 32 | HA 7 | GNSYPKLSKSYINDK |
| HA (A/California/04/2009) | 33 | HA 7 | PKLSKSYINDKGKEV |
| HA (A/California/04/2009) | 34 | HA 7 | KSYINDKGKEVLVLW |
| HA (A/California/04/2009) | 35 | HA 7 | NDKGKEVLVLWGIHH |
| HA (A/California/04/2009) | 36 | HA 8 | KEVLVLWGIHHPSTS |
| HA (A/California/04/2009) | 37 | HA 8 | VLWGIHHPSTSADQQ |
| HA (A/California/04/2009) | 38 | HA 8 | IHHPSTSADQQSLYQ |
| HA (A/California/04/2009) | 39 | HA 8 | STSADQQSLYQNADA |
| HA (A/California/04/2009) | 40 | HA 8 | DQQSLYQNADAYVFV |
| HA (A/California/04/2009) | 41 | HA 9 | LYQNADAYVFVGSSR |
| HA (A/California/04/2009) | 42 | HA 9 | ADAYVFVGSSRYSKK |
| HA (A/California/04/2009) | 43 | HA 9 | VFVGSSRYSKKFKPE |
| HA (A/California/04/2009) | 44 | HA 9 | SSRYSKKFKPEIAIR |
| HA (A/California/04/2009) | 45 | HA 9 | SKKFKPEIAIRPKVR |
| HA (A/California/04/2009) | 46 | HA 10 | KPEIAIRPKVRDREG |
| HA (A/California/04/2009) | 47 | HA 10 | AIRPKVRDREGRMNY |
| HA (A/California/04/2009) | 48 | HA 10 | KVRDREGRMNYYWTL |
| HA (A/California/04/2009) | 49 | HA 10 | REGRMNYYWTLVEPG |
| HA (A/California/04/2009) | 50 | HA 10 | MNYYWTLVEPGDKIT |
| HA (A/California/04/2009) | 51 | HA 11 | WTLVEPGDKITFEAT |
| HA (A/California/04/2009) | 52 | HA 11 | EPGDKITFEATGNLV |
| HA (A/California/04/2009) | 53 | HA 11 | KITFEATGNLVVPRY |
| HA (A/California/04/2009) | 54 | HA 11 | EATGNLVVPRYAFAM |
| HA (A/California/04/2009) | 55 | HA 11 | NLVVPRYAFAMERNA |
| HA (A/California/04/2009) | 56 | HA 11 | PRYAFAMERNAGSGI |
| HA (A/California/04/2009) | 57 | HA 12 | FAMERNAGSGIIISD |
| HA (A/California/04/2009) | 58 | HA 12 | RNAGSGIIISDTPVH |
| HA (A/California/04/2009) | 59 | HA 12 | SGIIISDTPVHDCNT |
| HA (A/California/04/2009) | 60 | HA 12 | ISDTPVHDCNTTCQT |
| HA (A/California/04/2009) | 61 | HA 12 | PVHDCNTTCQTPKGA |
| HA (A/California/04/2009) | 62 | HA 12 | CNTTCQTPKGAINTS |
| HA (A/California/04/2009) | 63 | HA 13 | CQTPKGAINTSLPFQ |
| HA (A/California/04/2009) | 64 | HA 13 | KGAINTSLPFQNIHP |
| HA (A/California/04/2009) | 65 | HA 13 | NTSLPFQNIHPITIG |
| HA (A/California/04/2009) | 66 | HA 13 | PFQNIHPITIGKCPK |
| HA (A/California/04/2009) | 67 | HA 13 | IHPITIGKCPKYVKG |
| HA (A/California/04/2009) | 68 | HA 13 | TIGKCPKYVKGGCG |
