# Supplementary material for: Enhanced Neutralizing Antibody Titers and Th1 Polarization from a Novel Escherichia coli Derived Pandemic Influenza Vaccine
Source: PLoS One. 2013 Oct 18;8(10):e76571. doi: 10.1371/journal.pone.0076571 (PMC3799843; doi:10.1371/journal.pone.0076571)
Supplement: Table S2 — Sequences of Qβ specific peptides used for re-stimulation of splenocytes from vaccinated mice. (DOCX) [file pone.0076571.s004.docx]

**TABLE S2 Sequences of Qβ specific peptides used for re-stimulation of splenocytes from vaccinated mice.**

| Protein | Peptide # | Peptide Cluster | Amino Acid Sequence |
| --- | --- | --- | --- |
| Qβ | 1 | Qβ 1 | AKLETVTLGNIGKDG |
| Qβ | 2 | Qβ 1 | TVTLGNIGKDGKQTL |
| Qβ | 3 | Qβ 1 | GNIGKDGKQTLVLNP |
| Qβ | 4 | Qβ 1 | KDGKQTLVLNPRGVN |
| Qβ | 5 | Qβ 1 | QTLVLNPRGVNPTNG |
| Qβ | 6 | Qβ 2 | LNPRGVNPTNGVASL |
| Qβ | 7 | Qβ 2 | GVNPTNGVASLSQAG |
| Qβ | 8 | Qβ 2 | TNGVASLSQAGAVPA |
| Qβ | 9 | Qβ 2 | ASLSQAGAVPALEKR |
| Qβ | 10 | Qβ 2 | QAGAVPALEKRVTVS |
| Qβ | 11 | Qβ 3 | VPALEKRVTVSVSQP |
| Qβ | 12 | Qβ 3 | EKRVTVSVSQPSRNR |
| Qβ | 13 | Qβ 3 | TVSVSQPSRNRKNYK |
| Qβ | 14 | Qβ 3 | SQPSRNRKNYKVQVK |
| Qβ | 15 | Qβ 3 | RNRKNYKVQVKIQNP |
| Qβ | 16 | Qβ 4 | NYKVQVKIQNPTACT |
| Qβ | 17 | Qβ 4 | QVKIQNPTACTANGS |
| Qβ | 18 | Qβ 4 | QNPTACTANGSCDPS |
| Qβ | 19 | Qβ 4 | ACTANGSCDPSVTRQ |
| Qβ | 20 | Qβ 4 | NGSCDPSVTRQAYAD |
| Qβ | 21 | Qβ 5 | DPSVTRQAYADVTFS |
| Qβ | 22 | Qβ 5 | TRQAYADVTFSFTQY |
| Qβ | 23 | Qβ 5 | YADVTFSFTQYSTDE |
| Qβ | 24 | Qβ 5 | TFSFTQYSTDEERAF |
| Qβ | 25 | Qβ 5 | TQYSTDEERAFVRTE |
| Qβ | 26 | Qβ 6 | TDEERAFVRTELAAL |
| Qβ | 27 | Qβ 6 | RAFVRTELAALLASP |
| Qβ | 28 | Qβ 6 | RTELAALLASPLLID |
| Qβ | 29 | Qβ 6 | AALLASPLLIDAIDQ |
| Qβ | 30 | Qβ 6 | ASPLLIDAIDQLNPAY |
